# Challenges and responsibilities in the medication management process in 24/7 group housing services for adults with intellectual disability: Interviews with nurses

**DOI:** 10.1177/17446295231163979

**Published:** 2023-03-18

**Authors:** Anne Halmetoja, Antti Teittinen, Raisa Laaksonen

**Affiliations:** Department of Pharmacology and Pharmacotherapy, Faculty of Pharmacy, 3835 F University of Helsinki, Helsinki, Finland; Research Department, The Social Insurance Institution of Finland, Nordenskiöldinkatu 12, 00250 Helsinki; Department of Pharmacology and Pharmacotherapy, Faculty of Pharmacy, 3835 F University of Helsinki, Helsinki, Finland

**Keywords:** Group homes, healthcare system, intellectual disability, medication management, nursing staff

## Abstract

Staff in 24/7 group housing services for adults with intellectual disability are responsible for ensuring safe medication management processes and supporting the residents in their health-related issues. Ten interviewed nurses reported several challenges in the medication management process emerging at the staff level, the level of the group home, and the level of the social and healthcare system, and were often related to issues in communication and responsibility. They reported a variety of complex tasks in the medication management process, for which they need a multiple skill set. They also act as healthcare advocates for residents, but healthcare services do not always match residents’ needs. Training for social and healthcare professionals, access to healthcare services and the collaboration of social and healthcare services should be improved to provide the people with intellectual disability the best possible pharmacotherapy and healthcare.

## Introduction

People with intellectual disability and/or autism who live in 24/7 housing services are in the need of constant support and help because of their functional and health challenges, which are only increasing as these persons are living longer than before (Patja et al. 2000, Schepens et al. 2019). The specialised healthcare services provided by the old long-term residential institutions are replaced with more general social care and healthcare services. There is evidence that the healthcare needs of people with intellectual disability are unmet after deinstitutionalisation (Ministry of Social Affairs and Health, 2016, Weise et al., 2017). The care staff of the housing services provide medication management and oversight and basic nursing care. The staff act with, or on behalf of, the residents when they use social care and healthcare services. Health issues often require pharmacological treatment but carrying out this treatment might be complicated by the residents’ functional challenges. The aims of this study were to explore from the care staff’s perspective what tasks and responsibilities they have, what skills and knowledge are required of them, and what kind of challenges the care staff have experienced in the medication management process.

## Background

Long-term residential institutions for people with intellectual disability have been abolished in many countries since the 1980s and 1990s (Mansell and Ericsson, 1996). In Finland, the process started in 2009 (Ministry of the Environment, 2009) and the goal was to deinstitutionalise the population with intellectual disability who lived in segregated residential institutions by 2020 (Ministry of Social Affairs and Health 2016, 2018). Institutional living has been replaced with municipal housing services, such as group housing, organised by municipal social care. The housing services should be tailored to match the individual needs (Social Welfare Act 1301/2014 21§).

The highest level of help and support in every day life is provided in 24/7 housing services for people with intellectual disability or autism, who need extensive help in day-to-day activities. These services include medication management and basic nursing care, but not necessarily medical care. The medical care services are provided mainly by primary care facilities (Ministry of Social Affairs and Health, 2016). In Finland, public primary care services are provided by municipal health centres. Private healthcare services are also available. Municipal outpatient disability clinics function under social care services and support the residents in issues related to mobility and financial aids, education, rehabilitation, therapy and living (The Act on Special Care for People with Intellectual Disabilities 519/1977). The municipalities organise the disability services in different ways. Thus, the medical services in some of these disability clinics are limited to intellectual disability diagnosing and rehabilitation plans, but some clinics offer also medical consultations for disability related issues. Since 2017, only electronic prescriptions are issued in Finland. The e-prescriptions are stored in a separate part of the Finnish Patient Data Repository, a centralised database containing personal healthcare records. Most prescriptions are valid for two years. The members of staff in the housing services, act or may act as patient representatives with, or on behalf of, the residents in healthcare related matters, but they cannot access the residents’ medical or prescription information in the Finnish Patient Data Repository.

Providing care for this patient group is challenging for primary care services (Ministry of Social Affairs and Health, 2016). Compared to people without intellectual disability, people with intellectual disability have 2.5 times more health related problems, in particular epilepsy and other neurological disorders, psychiatric disorders, musculoskeletal problems, and multiple congenital anomalies (van Schrojenstein Lantman-De Valk et al., 2000). Most people with intellectual disability have difficulties in communication, which are even more challenging if the person has a sensory impairment, such as hearing or visual impairment. Mental health conditions, sensory impairments and physical disability are even more common with co-occurring intellectual disability and autism (Dunn et al., 2020) and they add to the complexity in the diagnostic process and treatment. Multiple studies have raised the concern that the general primary care services do not match the needs of people with intellectual disability (Ministry of Social Affairs and Health, 2016; Weise et al., 2017).

Many of the health issues that are common in intellectual disability require pharmacotherapy (Straetmans et al., 2007). Compared to patients without intellectual disability, patients with intellectual disability use more medicines and their medication regimens may be more complex (O’Dwyer et al., 2018). For example, the dosing frequency per day might be higher, or there may be difficulties in giving medicines by routes of administration other than by mouth or there might be difficulties in adhering to special instructions required for administration of medicines, such as avoiding food when taking medicines, which may lead to a higher potential for medication-related problems (Erickson et al., 2017). When comparing people aged 50 years and over who had lived in the community, with or without intellectual disability, Peklar et al. (2017) showed that people with intellectual disability received more medicines and supplements and the drug treatment was more intense in the sense of excessive polypharmacy and extensive use of medications in epilepsy, mental health conditions and depression compared to people without intellectual disability. While people with intellectual disability are living longer than before (Patja et al., 2000), frailty begins (Evenhuis et al., 2012) and kidney function starts to decline (de Winter et al., 2014) earlier in people with intellectual disability. Frailty and renal insufficiency may increase sensitivity to medicines in ageing people (Lam et al., 2022; McLean and Le Couteur, 2004).

People who have intellectual disability often rely on others for help and support in the medication management process: in recognising the need for pharmacotherapy; consultations with physicians; purchasing the medicines; using the medicines as prescribed; and in monitoring the desired, and possible adverse effects (Erickson and Yang, 2019). Previously, the difficulties with medication management (Erickson and Yang 2019) and issues in the medication management process (Erickson et al., 2016) have been explored from the caregivers’ perspective, i.e., family, friends or employed support staff. In Finland, assisting patients and residents in medication management is considered a healthcare related task and should therefore be primarily performed by healthcare professionals in healthcare and social care services (Laukkanen and Ruokoniemi, 2021). Moreover, the employers in social care and healthcare are responsible for certifying the competence of their staff, including theoretical knowledge and practical skills of pharmacotherapy, at the beginning of employment and then every three to five years.

In Finland, registered nurses work only in residential services, where the residents have higher nursing care needs. If there is a registered nurse, s/he oversees the medication management process. The staff in residential services for people with intellectual disability are mainly registered practical nurses, Bachelors of Social Services, and assisting staff without any healthcare training, who do not always work under the supervision of a registered nurse. Where there is no registered nurse, the practical nurses have the overall responsibility of the medication management process.

The practical nurse training in healthcare and social care is a vocational upper secondary qualification of 180 competence points and usually takes 2,5 years of full-time studying. The practical nurses’ vocational qualification comprises eight elective competence areas, e.g., Care for the Disabled, Mental Health and Substance Abuse Work, and Nursing and Care. The extent and content of vocational pharmacotherapy training depends on the selected competence area, which means that newly qualified practical nurses have different readiness for the medication management tasks (Laukkanen and Ruokoniemi, 2021). Bachelors in Social Services can complete studies in pharmacotherapy during or after their vocational training. The extent and content of these studies vary as well and, compared to practical nurses, they lead to a lower competency. It is important that the medication-related competence of the staff matches their tasks and responsibilities, which might be more challenging in the 24/7 housing services, because of the resident profile and the possible problems related to the primary care services (Ministry of Social Affairs and Health, 2016; Weise et al., 2017). For clarity, in this article, all staff responsible for the medication management in the residential services are referred to as nursing staff to emphasise the healthcare role.

The aims of the study were:

1. To explore what tasks and responsibilities the nursing staff have in the medication management process in 24/7 group housing services for adults with intellectual disability, including the consultations with physicians, the acquisition, storage, dosing, and administration of medicines and monitoring the effects of individual pharmacotherapy of the residents;
2. To explore the nursing staff’s perspective on what skills and knowledge they are required to have in the medication management process in 24/7 group housing services for adults with intellectual disability; and
3. To explore the perceptions of nursing staff of the challenges in the medication management process in 24/7 group housing services for adults with intellectual disability.

## Methods

### Study design and location

This was a prospective qualitative study, employing individual semi-structured theme interviews of nursing staff working in 24/7 group housing services for adults with intellectual disability. Qualitative methodology was selected since rich information of personal views and experiences of nursing staff was required to achieve the aims of the study (Bowling, 2014). To promote transparency, this study is reported in line with the consolidated criteria for reporting qualitative research (COREQ) checklist for qualitative studies (Tong et al., 2007) (see Table S1).

In the recruitment of three 24/7 group homes for adults with intellectual disability and the participants, purposive, heterogeneous sampling was employed (Ritchie et al., 2003a). The group homes represented the three service provider types: one municipal; one private; and one of the third sector run by a trust. To ensure the group homes cannot be identified, the exact location of the study is not revealed, but all group homes operated in different districts of the same municipality in the capital area. The main investigator (AH) worked as a pharmacist in one of the group homes and her experiences gave the inspiration for this study.

The managers of the group homes were contacted first to seek their consent for the group homes to participate in the study; written consent was obtained. The group homes were offered an opportunity to discuss the general findings at the end of the study and to provide feedback on these findings. The investigator (AH) sent the managers recruitment letters and had online meetings with the managers to inform them about the study, to agree on practical issues around recruitment of staff and to collect background information about the group homes, e.g., resident profile, staff profile and general medication management arrangements. Even though all three group homes functioned 24/7, there were some differences in their resident profiles. Each of the group homes had around 10–20 residents. In one group home all residents had autism, but they were young adults with relatively few chronic physical illnesses. Another group home had residents of all adult age groups and with more service needs due to their chronic illnesses and physical disabilities. In these two group homes, the residents had their own rooms. In the third group home, the residents lived in their own apartments with 24/7 staff support. They were young or middle-aged adults with some chronic illnesses, such as diabetes or epilepsy. Psychotropic medicines, which are commonly used in the treatment of people with intellectual disability (Deutsch and Burket, 2021), were prescribed to residents in all group homes.

### Participants

The inclusion criteria for the participants were: (1) having the training of a nurse, practical nurse, or in social services with competence in pharmacotherapy; and (2) performing all medication-related tasks in the medication process. Altogether, the group homes had around 30 staff members meeting these criteria. With the help of the manager, the investigator (AH) had an online meeting with the staff of one group home to inform them about the study. Such a meeting could not be arranged with the other two group homes, but the other two managers forwarded an email containing a link to a video in which the investigator provided information about the study to the staff. The managers helped to distribute recruitment letters with study information to the staff. Additionally, in the weekly staff meetings in all three group homes, the managers informed the members of staff about the study in which they could opt-in to participate voluntarily and confidentially. They could participate in the interviews without their manager knowing and choose the time and the place for their interview. Written informed consent was obtained from each interviewee who returned the signed consent form in a prepaid envelope through mail. The participants were informed about the possibility to see the transcript of their interview and a café gift card of 10 euros was provided to each participant as a compensation for their time.

The goal was to recruit at least three participants from each group home, and after initial analysis, to recruit more participants until saturation point (Bowling, 2014), when no new information emerges in the interviews. After the initial recruitment, the investigator sent repeated recruiting emails to the staff through the managers. Before the interviews, the investigator phoned each consenting participant to collect the background information about their vocational training and working experience and addresses for sending the gift cards. Because of the Covid-19 pandemic, all contacts between the investigator and the participants had to be conducted by telephone, via emails and virtual online applications.

### Interview guide and visual stimulus material

**Figure 1.**
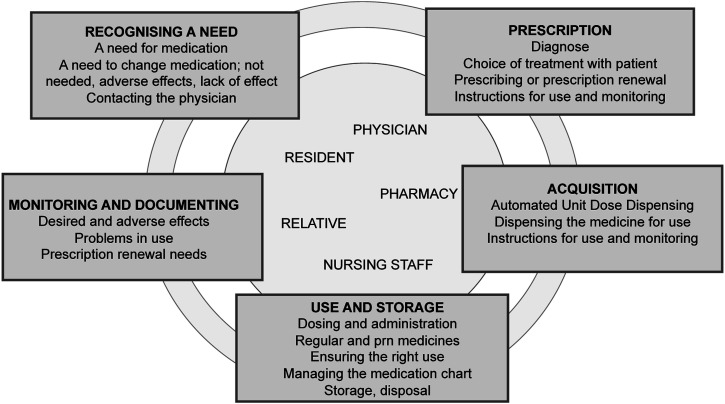
Graphic of the medication management process as used in the interviews as an activating element.

An interview guide and visual stimulus material in Finnish (Figure 1, translated into English), describing the medication management process, were developed based on the aims of the research, literature, the steps in the medication management process, background information about the group homes and the experiences of the research group. A pilot interview, which was not included in the analysis, was conducted with a volunteer practical nurse, whose mother tongue was not Finnish, to test the use of the interview guide and the intelligibility of the questions and the stimulus material. The pilot interview was also used to test the face and content validity of the interview guide (Lewis and Ritchie 2003). The guide and stimulus material were amended after the pilot interview.

### Data collection

Interviews were conducted individually to allow confidential discussions about personal perspectives and vocational experiences (Lewis, 2003). The interviews were conducted by the same investigator (AH) between January and May 2021, one by one, in the order of the received informed consents. The participants were shown the stimulus material of the medication management process early in the interview to activate them to talk about the entire process. All interviews conducted in Finnish were audio recorded. Field notes were not made. The lengths of the interviews varied between 45-72 minutes.

### Data handling and analysis

Every interview was transcribed verbatim in Finnish by the investigator (AH) as a way to familiarise herself with the data. The transcribed data were analysed inductively using the qualitative framework approach, which enhances the rigor of the analytical processes and the credibility of the findings (Ritchie et al., 2003b). During the analysis, the quotes were translated into English; RL checked the accuracy of the translations. AH had the main responsibility for the analysis while RL and AT checked the credibility of the analysis.

The data were first managed with Atlas.ti software (version 9.1.0). Initial categories were developed for the analysis after the first three interviews under the three main themes: 1) the tasks; 2) the required skills; and 3) the experiences. These categories were used as a coding matrix, which was refined throughout the data analysis. The data were coded and given descriptions and finally these descriptions were tabulated in a framework in a Microsoft Excel (version 16.55) spreadsheet, which enabled comparison between the participants. Then each category was broken into subcategories based on the data. The original transcripts were read repeatedly at every phase to ensure the descriptions were accurately created, coded, and categorised. Finally, explanatory descriptions were developed, interpreting, and comparing the descriptions in each subcategory. The investigators (AH, RL and AT) discussed, and agreed on, the directions of the analysis and the interpretations at every phase to ensure the trustworthiness of the analysis. Two of the group homes took the offered opportunity to hear a presentation about the general findings.

## Findings

Eleven signed informed consents were returned, and ten nurses were interviewed: four working at the private group home; four at the group home run by a trust; and two at the municipal group home. Three interviewees were registered nurses and seven were registered practical nurses. None of the Bachelors of Social Services participated. The investigator could not get in contact with one person to agree on the timing of the interview. The interviewees had different working experiences, in different health and social service areas and of different lengths. The practical nurses had completed studies in different competence areas. To preserve the anonymity of the participants, it is not possible to reveal further details of their background information. The participants were informed about the possibility to see the transcript of their interview but none of them took the opportunity.

**Figure 2.**
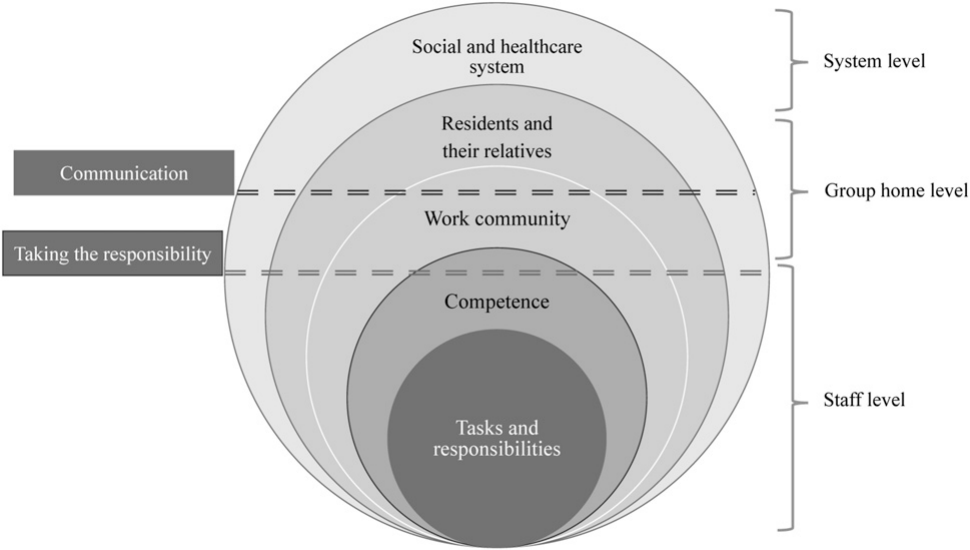
Levels and factors related to the challenges in the medication management process. Issues in communication and taking responsibility were common at different levels.

The interviewees discussed several challenges in the medication management process (Figure 2) at the staff level (the tasks, responsibilities, and competence of nursing staff), at the level of the group home (the work community, residents, and their relatives), and ultimately at the level of the social and healthcare system. Challenges in communication and in taking responsibility for the medication management process and the rational use of medicines of the residents were common to most levels.

### Challenges with the tasks and responsibilities

The nursing staff has the main responsibility for the medication management process of the residents. The process includes a variety of sequential tasks where the staff cooperates and communicates with each other, with other healthcare professionals outside the group home, and with the residents and their relatives (Table 1).

**Table 1.** The complexity of tasks of the nursing staff in the medication management process.

Starting the medicine	Acquisition, administration and handling of the medicine	Monitoring and evaluation of the effect of the medicine	Changing or discontinuing the medicine
**Recognizing the medical need** - Evaluation of the situation - Consulting colleagues - Cooperation with relatives - Documenting the evaluation **Contacting the physician** - Contacting the right physician - Documenting and reporting the contact - Cooperation with the relatives **Consultation appointment/call** - Accompany the resident - Support, advocacy and interpretation between physician and resident - Receiving physician’s orders: medicine, dosage, indication and length of use, monitoring - Documenting the orders - Managing and updating the medication list - Oral and written reporting of the order in-team - Cooperation with relatives	**Acquisition of medicines** - Ordering or collecting - Informing the pharmacy about changes in automated UDD^†^ - Monitoring the efficacy and adverse effects - Monitoring and documenting the ordering needs - Cooperation with relatives **Dosing and administration of medicines** - Automated UDD: checking and making modifications by hand - Dosing into weekly boxes, double checking, post-dosing modifications - Administering drops, sprays, injections etc. - PRN^‡^ medicines: evaluating the need, choosing the medicine, dosing, administration, evaluating the response - Checking the actions of the resident - Documenting the tasks **Storage and disposal of medicines** - Storage in the resident’s apartment/in the medication room - Monitoring the expiry dates - Monitoring the storage temperature - Monitoring and documenting the handling of medicines susceptible to abuse - Collecting the medication waste and sending it back to pharmacy	**Effects and actual use of the medicine** - Monitoring the problems in using medicines, such as swallowing problems - Documenting **Symptoms and behaviour** - Observation and evaluation - Cooperation with relatives - Consulting colleagues, documenting **Laboratory testing** - Asking for laboratory orders as scheduled - Booking the appointment - Accompany the resident or ordering a home testing service - If necessary, asking for authorization for physical restraint in advance - Making sure someone interprets the results - Receiving and documenting physicians’ instructions and making the changes to medication (acquisition, dosing) **Clinical testing in the unit** - Blood pressure or glucose, weight etc., regularly or when needed - Performing the testing as scheduled - Interpreting and documenting the result - Reacting to abnormal results	**Evaluating the need and effects** - Discontinuing the temporary medicine as scheduled - Detecting the medicines that are not needed anymore - Consulting colleagues - Cooperation with relatives **Contacting the physician, consultations** - Consulting a physician if indication is not clear, poor efficacy, adverse effects or other problems - Appointment or call - Receiving and documenting the physicians’ orders **Making and monitoring changes** - Updating the medication list - Making the changes to medication (acquisition, dosing) - Reporting the change in medication - Monitoring the change, including withdrawal symptoms

^†^UDD: unit dose dispensing,

^‡^PRN: “pro re nata” i.e., medicines used only when needed.

Although the final decision about starting, changing, or discontinuing a medicine is a responsibility of a physician, the nursing staff has an important proactive role in all this, since they often are the first to detect and evaluate the possible needs for medical checks of the residents and they arrange any required consultations with physicians.

The nursing staff is responsible for the internal medication management process in the group homes, including acquisition of medicines, their storage and disposal, dosing and administration, and monitoring of their effects. The participants perceived the routine tasks related to acquisition, storage and disposal of medicines, and dosing and administering medicines as the easiest tasks. On the other hand, they reported greatest challenges in the tasks that require interpreting a resident’s behaviour and symptoms or making contact with providers of medical care. Indeed, before administering “pro re nata” medicines (i.e., used only when needed), the nursing staff first evaluates the symptoms of a resident based on what the resident is able to communicate, or based on the resident’s behaviour, expressions, and utterances. They then decide if a medicine is the best solution to the needs of a resident.

Physicians plan and give instructions for monitoring the effects of each medicine they prescribe. The nursing staff is responsible for the monitoring based on observations about the resident. They evaluate their observations and report them to the physician if needed. The nursing staff is responsible for arranging any laboratory testing as referred by physicians and performing any clinical testing, such as blood pressure or blood glucose monitoring, in the group home.

One of the key tasks of the nursing staff is related to keeping the medication list of a resident up to date; both as an electronic version in the care management software of the group home and as a printed version, which is usually used when dosing or administering medicines to residents. However, for this task, the staff might receive information about the care plan only *orally* from physicians, during an appointment or a phone call, or even from a resident or a relative, when they have been in contact with a physician independently without a member of the staff. The interviewees saw this as a risk to medication safety and felt it would be crucial to obtain any medicines related information on *paper* during an appointment or at least via an email from the relatives to avoid mistakes.

Medical records and prescription information of patients treated in all public and private *healthcare services* in Finland are stored in the internal patient databases of the healthcare units and in the national Patient Data Repository. The residents and their family may have access to these Patient Data Repository records, but since the group homes provide *social care services*, the staff cannot access these records. As patient representatives of the residents, the staff may access information on electronic prescriptions only through a community pharmacy, if they have doubts about any prescribed medicines. However, e-prescriptions are up-to-date only if the prescribing physician has updated the e-prescription and not just recorded the information about a prescription on the internal patient database of the healthcare unit, which the community pharmacy and the staff at group homes cannot access. If the staff needs more information about a care plan of a resident, they must call the correct medical service provider, public or private, since the public and private service providers do not share information about the patients with each other.

### Challenges with the skills and competence

To perform all the medication related tasks, the nursing staff needs a variety of skills (Table 2). The staff needs to know the agreed practices in each task in the process, because the tasks are performed sequentially and usually by different staff members, in different shifts and on different days. The tasks should be performed flawlessly, documented well, and reported in the team to avoid hassles. To be able to act as an advocate for the residents, the nursing staff needs to know how the healthcare and social care services function for each resident; which social or healthcare provider, public or private, is involved and in which issues.

*“The mathematical skills are emphasised [in vocational practical nursing training], but the fact is that in the clinical practice, of course you need to know the basic calculations, but they should have more pharmacological knowledge.”*

*(Registered nurse 2)*

**Table 2.** The multifaceted set of skills and knowledge required from the nursing staff in medication management process and the methods to gain these skills and knowledge.

Starting the medicine	Acquisition, administration and handling of the medicine	Monitoring and evaluation of the effects of the medicine	Changing or discontinuing the medicine
**Recognizing the medical need**	**Acquisition of medicines**	**Effects and actual use of the medicine**	**Reviewing the need and effects**
- Knowing the resident’s normal patterns of behaviour, knowing when something is wrong	- Knowing the ordering and cooperation practices with the pharmacy, monitoring the ordering needs	- Knowledge of the medicines, especially the psychotropics used in the working unit	- Understanding the information on medication lists
- Knowledge on diseases, both acute and long-term and their symptoms	- Knowing the automated UDD^¶^ practises	• Therapeutic role in care	- Knowing the medicine and its’ therapeutic role
- Knowledge on the special aspects of behaviour and health in intellectual disability	*Methods to gain the skills:*	• Desired effect and how it is monitored	- Recognizing the possible needs to change or discontinue medication
*Methods to gain the skills:*	*Induction and learning on-the-job*	• The adverse effects and how they are monitored	*Methods to gain the skills:*
*Vocational training*	**Administration of medicines**	- Recognizing problems in medication use	*Vocational training and CPD activities*
*Learning on-the-job*	- Understanding the information on medication lists	*Methods to gain the skills:*	*Learning on-the-job, peer support*
*Personal studies or CPD* ^‡^ *activities on diseases and intellectual disability*	- Skills in dispensing and making changes to dispensed medicines, aseptically and flawlessly	*Vocational training and CPD activities*	**Contacting the physician, consultations**
**Contacting the doctor**	Administration skills:	*Learning on-the-job, peer support*	- Knowing the responsible physician for each resident / health issue
- Knowing the responsible physician for each resident / health issue	- Checking the ”five rights”: medicine, dose, time, route, client	**Symptoms and behavior**	- Knowing the best way to contact the physician
- Knowing the best way to contact the physician	- Non-invasive routes and subcutaneous injections	- Knowing the resident to recognize changes in symptoms or behaviour	- Language, documenting and reporting skills
*Methods to gain the skills:*	- In challenging situations, such as for agitated resident or in swallowing difficulties	*Methods to gain the skills:*	
*Induction and learning on-the-job*	*Methods to gain the skills:*	*Learning on-the-job, peer support*	*Methods to gain the skills:*
**Consultation appointment/call**	*Vocational training*	**Laboratory testing**	*Induction and learning on-the-job*
- Language skills	*Learning on-the-job, peer support*	- Knowing the practices to monitor the schedules, ask for orders and book testing	**Making and monitoring changes**
- Knowing what to ask or check	**Storage and disposal of medicines**	- Knowing the practices for blood sampling and interpreting/getting the results	- Knowing the practices required to make the change: acquisition, dispensing, disposal
- CMS^§^ skills: documentation and updating the medication list	- Knowing the right storage conditions	- Language skills	- Skills to monitor and manage the change, including withdrawal symptoms
- Efficient In-team informing and reporting skills	- Literacy of packages and labels	*Methods to gain the skills:*	*Methods to gain the skills:*
*Methods to gain the skills:*	- Knowing the storage and disposal practices	*Learning on-the-job, peer support*	*Vocational training and CPD activities*
*Induction and learning on-the-job*	*Methods to gain the skills:*	**Clinical testing in the unit**	
	*Vocational training*	- Testing skills (for example blood pressure or glucose)	*Learning on-the-job, peer support*
	*Induction and learning on-the-job*	- Skills to interpret the result and react accordingly	
		*Methods to gain the skills:*	
		*Vocational training*	
		*Learning on-the-job*	

^‡^CPD: continuing professional development,

^§^CMS; care management software,

^¶^UDD: unit dose dispensing.

Understanding the information on medication lists, including skills in mathematics, is required throughout the medication management process, for nurses to be able to update the medication list and to dose and administer medicines correctly. In addition, the nursing staff needs to have knowledge of medicines, their dosage forms and pharmacology, to handle medicines appropriately and safely and to monitor their desired effect and possible adverse effects. For the monitoring, they need to know the residents well to be able to notice a change in their behaviour or wellbeing and to evaluate the situation. Their competence is tested especially in challenging situations, such as when a resident refuses to take a medicine, is agitated or has swallowing problems or when a non-speaking resident’s behaviour changes dramatically.

The participants reported a gap between the readiness achieved through the vocational training and the skills required for the tasks and the responsibilities. Of the practical nurses, those who had completed studies in the Nursing and Care competence area, and those, who had completed their training through apprenticeships, were most content in their readiness in pharmacotherapy. The registered nurse and practical nurses with training or professional experience in mental health felt it was very useful for their current job, since psychotropic medicines are commonly used in their group homes. While the registered nurses had more healthcare training compared to practical nurses, they felt that their training had not prepared them to work with people with an intellectual disability or autism.

“*In my opinion, the theory comes from school. But then the practice, for example challenges in administration [of medicines] and such, of course they are difficult to learn at school.”*

*(Practical nurse 7)*

*“One must keep these [pharmacology related] things in mind all the time and recollect them. Pharmacotherapy is easily seen as merely dosing and administrating. That’s what it is for the most part, for me too.”*

*(Practical nurse 7)*

*“I think the job has been the best school for me. When you have the task list and diagnosis, it’s wonderful to read, to understand why, why this medicine, what is it, you know.”*

*(Practical nurse 6)*

The participants perceived that the competence gap between their theoretical learning and what is required in practice could be filled in several ways, but, in reality, it is often left as their own responsibility. Indeed, their employers had provided very little external pharmacotherapy training to them, apart from the online courses and exams they have to take every 3-5 years for a competence certificate in order to participate in the medication management process. However, the participants had not taken any courses on their own either, apart from one practical nurse, who also reported difficulties in finding suitable courses to take. The nursing staff felt that they need to be active to learn on-the-job and that the induction phase was important for ensuring their practical competence. The staff did a lot of peer teaching through helping and supporting each other. The participants, who had a pharmacist in their team, mentioned her as an important support in learning on-the-job. The participants mentioned psychotropic medicines most often as a topic they wished to have more training on. Most of them preferred face-to-face lectures over online courses.

### Challenges with the process in the group home and “in-team” cooperation

Most of the participants felt that the internal medication management process in their group home worked quite well, although errors do occur, for example in communication, in keeping the medication lists up to date, in the weekly dosing of medicines, or in noticing the need to order medicines from the pharmacy. The errors were seen as human errors related to inattentiveness in routine tasks or caused by unexpected situations. Ensuring that important information was communicated to everyone was complicated and time consuming:

“*We try to communicate [with each other] through the [care management software] messages and notes, we tell [the important things to each other], we write them in every single place, to make the information flow.”*

*(Practical nurse 6)*

In-team communication was reported to be essential for providing high quality services for residents but challenging to achieve. For example, when a physician calls after a call-back request, the responding staff member needs to know why the request was made to be able to communicate with the physician, for example to provide the right information about the right resident. Many of the practical nurses may have an immigrant background and their Finnish skills might be moderate. The language skills were reported to make communication challenging.

While the participants felt it was important for everybody to have the same responsibilities and duties in the medication process, some reported a mismatch because the assisting staff does not have any healthcare training, and thus, no responsibility of the medication process. The tasks in the medication management process take time, and if that is not taken into account in the distribution of tasks, time pressure and, sometimes, conflicts in the work community are created. The participants, who had a pharmacist in their team, mentioned her as having an important advisory and supportive role in the medication management process.

*“We have a very good and competent pharmacist, and we have a good collaboration. I can check these [medicines related] things with her and at least when we have gone through medication lists together, I’ve learned many new things about medicines.”*

*(Registered nurse 1)*

### Challenges related to residents and their relatives

The participants felt it is important to allow the residents do everything they are able to do, but this creates also challenges, since the nursing staff is responsible for the entire medication management process, even when residents act on their own and the staff is not witnessing their actions, for example the self-administration of medicines. Thus, the staff cannot know for sure what the resident actually did or did not do, and so, they felt it is impossible for them to bear the responsibility.

In every group home, some residents had difficulties in swallowing or a tendency to chew tablets and capsules. To ease the swallowing, the nursing staff cuts or crushes tablets, which can compromise the efficacy or safety of the pharmacotherapy. The residents’ motor and cognitive disabilities lead to several challenges in the medication management process or even situations, where the residents are not receiving the medicines they need.

*“So sometimes we crush the medicines and it’s not always good, and not even allowed necessarily. And then quite often, when the swallowing problems are a major issue, the physicians just discontinue the medicines”*

*(Registered nurse 2)*

*“A resident had a fungal nail infection and should have had a course of medicines for it, but couldn’t take the whole course, because there weren’t other forms of it than by mouth.”*

*(Practical nurse 2)*

Some residents had difficulties in accessing health centres, because of pronounced anxiety in strange surroundings or being bed bound. Since the public healthcare does not provide physician house calls, these residents do not receive medical care if they cannot afford to use private medical services. The participants also talked about the challenges in performing physical examinations because, for example, a resident would not allow a physician to come close, or the health centre did not have a hoist for a non-mobile resident.

The residents’ cognitive disability and communication problems mean that they have trouble in recognising and communicating changes in physical and mental health. The nursing staff has to use other cues, such as abnormal behaviour and expressions. Participants reported this to be difficult, but that it becomes easier, when they get to know the residents. The staff also talked often with each other about their observations, to validate them and to reach a shared opinion about the actions to take.

The participants reported challenges in communication, in sharing the responsibility and tasks, and disagreements, if the residents’ relatives take part in the medication management process. There might be confusion about what has been prescribed to the resident and what over-the-counter products the resident uses. Relatives might have strong opinions about what medicines a resident should use, but the nursing staff always follows the physicians’ orders. The relatives might have an influence in what the physicians prescribe, and the result might not be what the nursing staff feels is best for the resident. The care plan information might come to the staff only from a relative, not from the physician. Thus, the participants preferred bearing the whole responsibility for the medication management instead of sharing it with the relatives.

### Challenges with the social and healthcare system

When the residents have minor problems with their physical health, the participants reported the municipal healthcare services to be satisfactory. If the disability related problems are quite stable, and intensive or drastic interventions are not needed, the physicians’ services from outpatient disability clinic work well. When a resident has more or greater needs and especially if a resident has mental health issues, things were perceived to become more difficult. As the residents’ healthcare advocates, the participants felt it is their responsibility to make sure the residents get the help they need from the social and healthcare system, so these difficulties were reported to be emotionally distressing and frustrating. The first problem might come when physicians in both the health centre and the disability clinic refuse to take responsibility a resident’s health related issue. The participants reported having to call both organisations several times and “fight” with the system before finding a physician to help. The service paths and referral times were reported to be long, so getting help might take several months, during which time the staff is worried about the wellbeing of the resident, the resident’s health might worsen, and the resident’s behavioural problems might have a negative effect on other residents as well, causing distress, conflicts and even violence between residents and towards staff.

“*People with intellectual disability need an expert, who takes care of them and understands their needs. Not in a health centre, in my opinion.” (Practical nurse 6)*

The health centres were reported not to be equipped to serve residents with severe autism or intellectual disability. The physicians change often, and they don’t have expertise or experience in intellectual disabilities and autism. Some participants had experienced situations, where the physician got confused or wanted to get quickly rid of the resident, because the resident had intellectual disability or autism. Physicians might talk only to a member of staff, when a resident could speak for him or herself, or try to talk with a non-speaking resident, even when told that the resident does not speak. As reported earlier, the centres might lack required equipment to serve disabled people, such as hoists, and are not even accessible for all residents. The group homes used health centres of three districts of the same municipality, and the services were reported to be different while they should be the same. For example, one group home had a contact nurse in their health centre who called the group home once a week, and this was not the group home in which the residents had the most medical needs. The other group homes did not have such a service. Hospice care for a dying resident was easily arranged through home hospital in one district but had been very difficult in another because the resident had intellectual disability. On the other hand, the participants were very content in community pharmacy services.

The participants gave several suggestions on how the medical services could be enhanced. First, they wish the services would be tailored to the resident’s individual needs. The health centre should have a physician or a contact nurse with expertise in autism and intellectual disability. Some wished that the outpatient disability clinic would take responsibility for all the healthcare needs of a person or that there would be a special health centre for people with intellectual disability, with all specialists under the same roof. It would be important for some residents, and for others in certain situations, to have a physician, who could make house calls when needed, or regular rounds in the group home, similarly to geriatric residential services.

## Discussion

The present study is the first to explore the perceptions and experiences of nursing staff about the medication management process in 24/7 group housing services for adults with intellectual disability. The nursing staff have the main responsibility for the medication management process of the residents, where they have various, and complex tasks. They also act as advocates for the residents in the social care and healthcare system (see also Brolan et al., 2012). The registered practical nurses are healthcare professionals, but their vocational training often gives them only a partial readiness for these tasks and responsibilities. In addition, the social care and healthcare system does not always provide them, or the residents, the support they need.

While in this study all participants were healthcare professionals and had better readiness for pharmacotherapy, the findings were similar to the findings of a previous study on perceptions of caregivers (i.e., family and staff) supporting people with intellectual disability at a family home or in a group home by Erickson et al. (2016). The similarities could be observed in issues occurring at the diagnosis and prescribing phase (prescription information, communication, and interaction), in administering medication (refusal to take medication, palatability of medication), in monitoring therapy (difficulty identifying side effects of medications, need for additional training) and in using the healthcare system (unpreparedness of the healthcare system, lack of care coordination, and lack of integration of the information).

Medication is often an important part of the care or treatment of many health problems, but only if it is provided at the right time, as the right medication and in the right manner (World Health Organisation, 2022). The challenges the participants reported in obtaining a medical consultation for the residents are alarming and implicate a risk of people being left without medical care or medicines, which is against the principles of Article 25 in the United Nations’ Convention on the Rights of Persons with Disabilities. Another common obstacle the participants mentioned was the lack of suitable formulations of medicines for people with swallowing problems, resulting in not receiving any, or receiving suboptimal, treatment.

In addition, the errors and challenges in the medication management process can compromise the safety of the residents’ pharmacotherapy. The systems approach to human error (Reason, 2000) states that errors occur because of the conditions under which the individuals work. Latent conditions that cause errors or predispose to them include complexity, communication, competence and experience, variation, allocated working time, tiredness, and stress. The participants reported active failures or unsafe acts such as slips, lapses, fumbles, mistakes, and procedural violations, but also a lot of complexity and variation, and challenges in communication in the medication management process in these group homes, both internally and in the collaboration with the relatives and the social care and healthcare service system. The staff might not always have the required competence or experience and they face time pressure in medication-related tasks. These factors make the process vulnerable and prone to errors, but research of medication safety in 24/7 housing services for people with intellectual disability is still scarce.

The importance of the competence of staff in the medication management process and in pharmacotherapy for medication safety is highlighted in the latest national guidance in Finland (Laukkanen and Ruokoniemi, 2021). As expected, the participants perceived that the content and extent of pharmacotherapy teaching in vocational training varied, especially for practical nurses. Mostly, the responsibility of post-graduate learning is left to the staff; to individual activity and to peer or in-team learning and teaching. For the in-team competence to be adequate, there should be enough healthcare professionals in the staff, despite also having assisting staff. In addition, it is best to have multidisciplinary care teams with not only practical nurses but also registered nurses and pharmacists with their complementary skills and knowledge, since interprofessional collaboration leads to better care (WHO 2010). Pharmacists are still an underused resource in services for people with intellectual disability (Lee et al., 2021), compared to geriatric services, for example. However, a recent scoping review revealed several types of pharmacist interventions that could benefit the medication management of persons with intellectual disabilities (Lee et al., 2021); these varied from the traditional role of dispensing medicines to educational and advisory roles, roles in general medication management and roles in multiprofessional teams. Thus, in 24/7 housing services pharmacists could have several tasks and roles in promoting and developing safe medication practices, in reviewing the residents’ medication regimens and as an educational and advisory support for other professionals, residents and their relatives.

The participants perceived that the induction phase had been important for them to learn the tasks and practicalities of the medication management process, but also to get to know the residents, their abilities, challenges, and behavioural patterns. Building a relationship with the residents takes time. It is important that the members of staff do not change too often, for the staff as a whole to retain these relationships and the knowledge about the residents’ individual abilities and needs. Stress affects work satisfaction and workforce stability (Van Bogaert et al., 2013). Work dissatisfaction and job strain have been reported as factors associated with the intentions of staff to change jobs in services for people with intellectual disability (Hatton et al., 2001). Several stress factors were reported in this study, such as too many tasks compared to working time, challenging behaviour of the residents and the fact that the surrounding social and healthcare service system does not always give help and support but needs to be “fought with”.

These findings can be used to indicate how to develop and promote the safety and efficiency of the medication management process and the competence of the staff in 24/7 group housing services for adults with intellectual disability: (1) The pharmaceutical industry should pay more attention to the formulation and the palatability of medicines so that people with intellectual disability would be able to use the medicines they need (see also Robertson et al., 2017); (2) The basis of professional competence is in vocational training. For professionals aiming to work with people with intellectual disability, autism or both, the training should go beyond the basic skills of dosing and administering, including a more thorough understanding of pharmacotherapy, especially psychotropic medicines, administration of medicines in challenging situations, and monitoring the desired and adverse effects (see also Erickson and Yang, 2019); (3) Both staff and their employers are responsible for the post-graduate learning. Employers should be active to provide courses and promote training activities through allocating working time for studying. Post-graduate training providers should take into account the learning needs of the staff working in housing services for people with intellectual disability; (4) Employers should make sure the staff has enough allocated working time for the orientation, and for the medication related tasks that are numerous and can be time consuming. The expertise of pharmacists should be utilised more since they can help and support the medication management process in many ways (see also Lee et al., 2021); and (5) People with intellectual disability, autism or both have special healthcare service needs that should be taken into account in the training of all healthcare professionals and in the provision of healthcare services to make them accessible in every way: the premises and their selection of equipment and supplies; providing medical care at home when needed; and in the communication with the patients or their advocates (see also (O’Dwyer et al., 2018). Providing the same healthcare services for all citizens, with or without intellectual disability, can lead to neglecting the special needs of some people with intellectual disability. The services should be accessible for and tailored to match the individual needs of people with intellectual disability.

### Strengths and limitations

This was the first study to explore the perceptions and experiences of nursing staff about the medication management process in 24/7 group housing services for adults with intellectual disability. To the best of our knowledge, the tasks, responsibilities, and competence requirements in the medication management process have not been studied in such detail before. The group homes represented all three different types of housing service providers and had diverse resident profiles. All group homes of this study are located in the same municipality which allowed the comparison of differences in healthcare services even when the healthcare service organiser is the same. While it was challenging to recruit participants and the number of interviewees was low, the participants had different backgrounds (training, age, and work experience). Some of the participants knew the investigator AH personally but we believe this only made the interview situation easier for them and did not have great influence on their responses. Conversely, the participants who did not know the investigator might have felt less inclined to please the interviewer (Coar and Sim, 2006). On the whole, the interviews gave a rich data, and a saturation point was reached. RL and AT are experienced in qualitative research and AH has clinical experience from residential services for people with intellectual disability. The healthcare services might be organised in different ways in other parts of the country and internationally, but we feel that these themes derived from the analysis are common and transferable to, and applicable in, other districts and countries. The results are in line with previous studies of caregiver perceptions (Erickson et al., 2016), which support the transferability of the findings.

## Conclusion

The registered nurses and practical nurses working in adult 24/7 housing services for people with intellectual disability have an important and responsible shared role in the internal medication management process of the group homes, but they also act as advocates for the healthcare needs of the residents. The registered nurses and practical nurses face several challenges in the medication management process that relate to the tasks and responsibilities, their work community, the residents and their relatives, and the social care and healthcare service system. Their tasks and responsibilities require knowledge and several types of skills that should be addressed in their professional training and in supporting their learning and development at work. One of the major issues of concern relate to the healthcare services, especially in the mental health services, that do not always match to the needs of the residents and leave the registered nurses and practical nurses in frustration. Further research is needed to better understand what the root cause of these challenges is; management, lack of workforce or structural issues.

## Supplemental Material

Supplemental Material - Challenges and responsibilities in the medication management process in 24/7 group housing services for adults with intellectual disability: Interviews with nurses Supplemental Material for Challenges and responsibilities in the medication management process in 24/7 group housing services for adults with intellectual disability: Interviews with nurses by Anne Halmetoja, MSc, Antti Teittinen, PhD and Raisa Laaksonen, PhD in Journal of Intellectual Disabilities
